# "Person-in-the-Barrel" Syndrome: A Case Report of Bilateral Arm Paresis Due to Vasculitis With a Review of Pathological Mechanisms

**DOI:** 10.7759/cureus.13607

**Published:** 2021-02-28

**Authors:** Hassan Kesserwani

**Affiliations:** 1 Neurology, Flowers Medical Group, Dothan, USA

**Keywords:** small vessel vasculitis, inflammatory neuropathy, clinical and functional anatomy

## Abstract

"Person-in-the-barrel" syndrome is a descriptive term for bilateral arm (brachial) paresis in the absence of lower extremity (crural) weakness or bulbar (medullary) weakness. This phenomenon is associated with various descriptive terms such as "distal field infarction", "flail limbs", and "cruciate paralysis". Arriving at a specific diagnosis is a fascinating exercise in anatomical localization. Strategic lesions involving the watershed zones of the motor frontal lobes and the pyramidal decussation at the cervico-medullary junction are the classic sites of injury. However, peripheral causes such as motor neuron disease, mononeuritis multiplex (vasculitis), bilateral brachial plexopathy, and critical illness myopathy have been sporadically reported and can stochastically inflict the motor nerves or muscles of the upper extremities. In this report, we present a case of vasculitis with weakness restricted to the upper extremities and also delve into the neuropathological mechanisms of "person-in-the-barrel" syndrome.

## Introduction

"Person-in-the barrel" syndrome is a descriptive term for bilateral arm paresis in the absence of bulbar or lower extremity weakness. In order to understand the genesis of exclusive bilateral upper extremity weakness, one needs to understand the topography of the corticospinal tracts as they descend from the Betz cells, which are some of the largest cells in the central nervous system and located within the fifth layer of the primary motor cortex; they undergo about a 90-degree spiral torsion as they descend through the centrum semiovale, the corona radiata, the internal capsule, the basis pontis until the pyramidal decussation above the level of the foramen magnum, at the cervico-medullary junction. Lesions along this pathway that differentially involve the fibers to the upper extremities have been described using explicit terms such as "distal field infarctions" for watershed infarcts involving the cortex and "cruciate paralysis" for cervico-medullary lesions [1]. Other synonyms include "flail limbs", which is usually, but not always, reserved for cases of motor neuron disease and brachial diplegia [2].

Cases of cerebral hypoperfusion and watershed infarcts, cerebral metastases, pontine infarcts, central pontine myelinolysis, cervical spinal cord injury or trauma, and anterior spinal cord infarcts have been well documented [3-8]. However, lesions of the peripheral motor nerves of the upper extremities can also lead to exclusive upper extremity weakness. Myasthenia gravis, motor neuron disease (flail limbs), bilateral brachial plexopathies, and critical illness myopathy have also been described sporadically [9-12]. The pathogenesis underlying the differential selection exclusively of the upper extremities in these peripheral nerve disease problems is not fully understood. In the Discussion section of this article, we hypothesize on the variance of the energetics of the size of motor units and statistical stochastic processes.

"Person-in-the barrel syndrome" was formerly known as "man-in-the-barrel syndrome". Soares et al. have cogently recommended the former, gender-neutral term to accurately reflect the fact that the condition affects both genders; for instance, in their review involving 95 patients, 33% were female [2].

## Case presentation

We discuss the case of a right-handed 65-year-old-man who presented with progressive bilateral arm weakness of one year's duration. The weakness had initially started with an acute right wrist, finger, and thumb drop after pulling on a rope; at that time, he had felt a sharp pain in his right forearm and tingling over the radial forearm. Three months after that event, he underwent a right radial nerve decompression surgery at the arcade of Frohse (supinator muscle) with partial improvement. At eight months, he developed a left finger drop with contractures at the metacarpophalangeal joints and left elbow extension weakness. At that point, the weakness was potentially attributed to cervical cord compression, and a C4-C7 anterior cervical decompression with fusion was performed without improvement. By the 12th month, his condition had worsened to the point that he was unable to form an adequate grip to open a door, pronate or supinate his forearms, or raise his arms to shoulder level.

His past medical history was significant for idiopathic pulmonary fibrosis, for which he had consulted a pulmonologist. He was also being treated by a hematologist for an immunoglobulin M (IgM)-kappa monoclonal protein without any sclerotic bone lesion, plasmacytoma, or multiple myeloma. This monoclonal protein had been diagnosed as a benign monoclonal gammopathy of undetermined significance (MGUS).

The pertinent examination findings are listed below. Power in the upper extremities based on the Medical Research Council (MRC) grading was rated as follows: revealing profound bilateral arm weakness with diffuse bilateral arm atrophy with wasting of the thenar muscles of the hands. No fasciculations were visible (Table 1).

**Table 1:** Power grading of upper extremity muscles based on the Medical Research Council (MRC) guidelines

	Right	Left
Deltoids	1	1
Biceps	1	1 with contracture
Triceps	1	1 with contracture
Brachioradialis	2	1
Pronator teres	2	2
Supinator	2	2
Wrist extension	0	0
Wrist flexion	1	1
Finger extension	1 with contracture	1 with contracture
Finger flexion	1 with contracture	1 with contracture
Finger spreaders	1	1

There was areflexia of all the deep tendon reflexes of the upper extremities. The sensation was preserved to vibration in the index fingers bilaterally, with the absence of pseudoathetosis. No dermatomal demarcation of sensory loss to pin-prick was discernible except for diminished pin-prick over the deltoids bilaterally. However, there was reduced pinprick and light touch in the fingers. Bilateral lower extremity power and deep tendon reflexes were normal.

A nerve conduction study/electromyogram (NCS/EMG) of the upper extremities revealed bilateral absent ulnar, median, musculocutaneous, and superficial radial sensory nerve action potentials (SNAP). Bilateral median motor amplitudes were severely reduced with mildly diminished velocities. The right ulnar motor amplitude was moderately reduced and the left ulnar motor amplitude mildly reduced, with mildly reduced velocities. The radial motor amplitudes were bilaterally absent. An EMG revealed acute-on-chronic denervation in a patchy pattern involving the bilateral median motor nerves with varying degrees of severity (severe), bilateral ulnar motor nerves (severe), bilateral radial motor nerves (moderately severe, left worse than right), and bilateral axillary motor nerves (moderate severity, left worse than right). This pattern is consistent with a severe acute-on-chronic mononeuritis multiplex.

The cerebrospinal fluid (CSF) analysis was non-inflammatory with normal white cell count and protein. Laboratory studies included a full vasculitis/connective tissue disease; a battery of 35 serological tests revealed four abnormalities listed below; of note, an anti-myelin-associated glycoprotein (MAG) antibody was absent. This pattern is consistent with an autoimmune inflammatory process (Table 2).

**Table 2:** Abnormal serology findings PM/Scl: polymyositis/scleroderma

Variables	Results	Normal range
C-reactive protein	42.16	0-10.90 mg/L
Erythrocyte sedimentation rate	51	0-10 mm/hr
Quantitative rheumatoid factor	24	<14 international units/ml
Anti-PM/Scl antibody	34	<20

With the laboratory values indicating an inflammatory etiology and the electrophysiological study suggesting a mononeuritis multiplex, the patient was treated empirically with intravenous methylprednisolone 500 mg weekly for four weeks, followed by intravenous immunoglobulin (IVIG) 1 g/kg daily for two days, once a month for three months with 1-2 point improvement in MRC power grading of bilateral shoulder abduction, pronation/supination of the forearms, elbow flexion and extension, and wrist flexion and extension and finger spreaders, representing a significant improvement. 

Due to the initial focal presentation (wrist, finger, and thumb drop) followed by multi-focal involvement of the peripheral nerves (motor and sensory), absence of CSF inflammation [with chronic inflammatory demyelinating polyneuropathy (CIDP) being unlikely] but elevated serological inflammatory markers, a provisional diagnosis of mononeuritis multiplex (vasculitis) was made. Treatment with oral prednisone at a dose of 1 mg/kg with a slow taper and oral azathioprine with a target titration dose to 2 mg/kg, with an aim to maintain a mean corpuscular volume (MCV) greater than 100, was initiated. The macrocytosis is a surrogate marker of bone marrow suppression and an indicator that azathioprine is working. The patient is being carefully monitored. A superficial radial sensory nerve biopsy was not performed.

## Discussion

The topographical disposition of the corticospinal tract fibers to the arms, facial, bulbar muscles, and legs in relation to each other is important in trying to understand the preferential affliction of the motor fibers destined to the arm muscles. A brief overview of the pathophysiological mechanisms as reported in a few studies is outlined below (Table 3).

**Table 3:** Classification of the pathological anatomy of "person-in-the-barrel" syndrome

Study	Descriptive term	Anatomical localization	Mechanism
Orsini et al. [13]	Distal field infarction	Borderzone between the anterior and middle cerebral artery	Watershed zone corresponds to the motor homunculus of the proximal upper limbs; injury usually due to cerebral hypoperfusion, watershed infarcts
Laubscher et al. [14]	Cruciate paralysis	Cervico-medullary junction/pyramidal decussation	Corticospinal tract fibers of upper extremities descend more medially and anteriorly at the cervico-medullary pyramidal decussation above the level of the foramen magnum one cord segment above the level of lower extremity decussation. Usually traumatic
Orsini et al. [15]	Flail limbs	Motor nerves of upper extremities	Motor neuropathy due to motor neuron disease or vasculitis or inflammatory demyelination. Predilection is unexplained or stochastic

As illustrated in the table below, the corticospinal tracts descend from the Betz cells of the motor cortex through the corona radiata, internal capsule, midbrain, basis pontis down to the pyramidal decussation, and undergo an approximate 90-degree spiral torsion, displacing the arm representation from a distal location on the motor strip of the homunculus to an anteromedial location at the cervico-medullary junction, in relation to the fibers of the legs (Table 4) [1,16,17].

**Table 4:** Topography of corticospinal tract and its torsion as it descends from the frontal cerebral cortex to the pyramidal decussation

	Face	Arm	Leg
Cortex frontal lobe	Proximal	Distal	Medial
Corona radiata	Anterior	In-between	Posterior
Internal capsule	Anterior	In-between	Posterior
Midbrain - crus cerebri	Medial	In-between	Lateral
Basis pontis	In-between	Anteromedial	Posterolateral
Pyramidal tract	Exited to bulbar muscles	Medial	Lateral
Cervico-medullary junction	Exited	Anteromedial	Posterolateral

One can speculate that the motor nerves of the upper extremities are more dexterous (smaller motor units) than those of the lower extremities, and it may be that in those genetically predisposed, the smaller motor units are differentially affected. There is some merit to this theory as the small motor units are the slow-twitch-aerobic, high-mitochondrial-density, ATP-dependent pathway and may be more sensitive to inflammation and disruption of the metabolic activity [18]. However, this is purely hypothetical at this point and does not explain the involvement of the proximal muscle groups like the deltoids and biceps muscles. Another possibility is purely statistical: biological systems follow a normal distribution, and the bilateral brachial paresis patients could be the outliers on the tail of a normal distribution; hence, those affected are subject to a random or stochastic process. In simpler terms, the inflammatory process can affect the peripheral nerves of the arms and legs equally, arms more than legs, legs more than arms, the arms unevenly, the legs unevenly, and so on. There are many different permutations and combinations. The distribution is usually a normal distribution and the less likely or outlier possibility is the exclusive involvement of the arms. Herein lies the idea of randomness and the exclusive involvement of the arms being one of the rarer presentations. This is a biological fact just as the odds of finding people taller than seven feet is a rare phenomenon.

**Figure 1:**
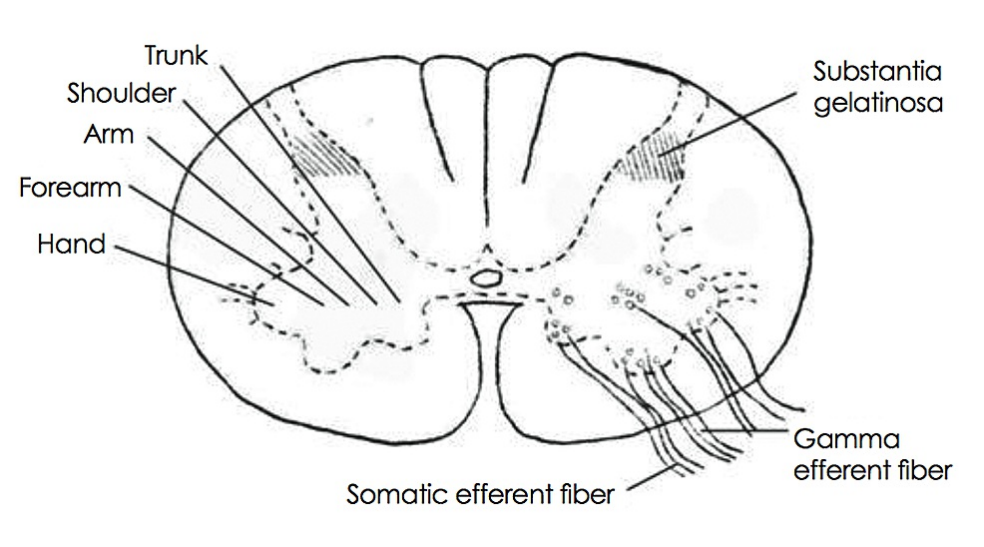
Topography of hand area in the anterior horn of spinal cord explaining the predisposition to anterior spinal cord ischemia

We have previously described a case of an epiconus syndrome with bilateral leg paresis and hypothesized that the weakness arises from the centrifugal location of the corticospinal tract fibers [19]. In contrast, Kim et al. hypothesized on the centrifugal location of the anterior horn cells (destined to the hands) in the anterior horn of the spinal cord, where anastomoses between circumferential and sulcal arteries are sparse, explaining the bilateral brachial weakness of their patient, due to the watershed effect (Figure 1) [20].

## Conclusions

"Person-in-the barrel syndrome" is a fascinating condition that can, in the majority of cases, be explained by the approximate 90-degree torsion of the corticospinal tracts as they descend from the motor cortex to the pyramidal decussation. The etiologies can be central or peripheral involving the cascade from the Betz cells of the motor cortex to the anterior horn cells of the spinal cord to the brachial plexus, peripheral nerve, neuromuscular junction to the very muscles themselves. We presented a very rare case of vasculitis (mononeuritis multiplex) presenting with bilateral arm paresis. This case report highlights the fact that initial neurologic symptoms consistent with mononeuritis multiplex must be carefully followed, as a subset of these patients may eventually deteriorate due to a more insidious disease with a greater disability and hence may require a more detailed evaluation and care. Though rare, understanding the anatomy involved in the pathogenesis of this phenotype is of utmost importance to the practicing clinician.
